# Modulation and Control of Wettability and Hardness of Zr-Based Metallic Glass via Facile Laser Surface Texturing

**DOI:** 10.3390/mi12111322

**Published:** 2021-10-28

**Authors:** Qinghua Wang, Yangyang Cheng, Zhixian Zhu, Nan Xiang, Huixin Wang

**Affiliations:** 1 School of Mechanical Engineering, Southeast University, Nanjing 211189, China; qinghua-wang@seu.edu.cn (Q.W.); zxzhu@seu.edu.cn (Z.Z.); nan.xiang@seu.edu.cn (N.X.); 2 Jiangsu Key Laboratory for Design and Manufacture of Micro-Nano Biomedical Instruments, Southeast University, Nanjing 211189, China; 3 Guangdong University of Science and Technology Coordination and Innovation Research Institute, Foshan 528000, China; ggdcnc@163.com; 4 Institute of Agricultural Facilities and Equipment, Jiangsu Academy of Agricultural Sciences, Nanjing 210014, China

**Keywords:** laser surface texturing, wettability, hardness, Zr-based metallic glass

## Abstract

Bulk metallic glass (BMG) has received consistent attention from the research community owing to its superior physical and mechanical properties. Modulating and controlling the surface functionalities of BMG can be more interesting for the surface engineering community and will render more practical applications. In this work, a facile laser-based surface texturing technique is presented to modulate and control the surface functionalities (i.e., wettability and hardness) of Zr-based BMG. Laser surface texturing was first utilized to create periodic surface structures, and heat treatment was subsequently employed to control the surface chemistry. The experimental results indicate that the laser textured BMG surface became superhydrophilic immediately upon laser texturing, and it turned superhydrophobic after heat treatment. Through surface morphology and chemistry analyses, it was confirmed that the wettability transition could be ascribed to the combined effects of laser-induced periodic surface structure and controllable surface chemistry. In the meantime, the microhardness of the BMG surface has been remarkably increased as a result of laser surface texturing. The facile laser-based technique developed in this work has shown its effectiveness in modification and control of the surface functionalities for BMG, and it is expected to endow more useful applications.

## 1. Introduction

Bulk metallic glass (BMG) has received considerable attention from the research community during the past several decades since its first discovery in the 1990s, mainly owing to its superior mechanical and physical properties [1], including high values of yield strength [2], high hardness [3], relatively low Young’s modulus [4], good corrosion and wear resistance [5], as well as excellent magnetic properties [6]. This gives BMG a variety of potential applications in the fields of bioimplants, magnetic materials, structural materials, sensors, microelectromechanical systems (MEMS), and micro/macro devices [7,8].

Besides its intrinsic outstanding physical and mechanical properties, researchers have also attempted to modify the surface functionalities of BMG, which can be achieved by introducing micro/nanostructures into BMG. The typical fabrication methods of surface structuring include magnetron sputtering [9,10], electro-oxidation [11], thermoplastic shaping [12,13,14], and laser surface texturing [15,16]. Among all the existing surface modification methods, laser surface texturing has demonstrated its strong potential as a highly efficient and cost-effective approach due to several key advantages including process efficiency, flexibility, ease for automation, and environmental friendliness [17]. 

In recent years, laser-based surface texturing has been proved to be one of the most efficient techniques to modify and control important surface functionalities, including surface wettability [18,19,20,21,22], reflectivity [23,24], anti-icing property [25,26], corrosion resistance [27], etc. For example, wettability transition from superhydrophilicity to superhydrophobicity has been achieved on various materials including aluminum [28], copper [29], stainless steel [30,31], and titanium [32,33,34,35,36] by combining laser surface texturing and low-temperature annealing. In terms of laser texturing for BMG materials, there have been some recent research efforts on modification of surface properties of BMG via creation of laser-induced surface texture and change of surface chemistry [15,16,37,38,39,40,41,42]. Huang et al. utilized nanosecond pulsed laser irradiation to fabricate hierarchical micro/nanostructures on the Zr-based metallic BMG substrate in order to increase the effective surface area [16]. The laser-modified BMG surface retained amorphous characteristics, and the elemental distribution on the surface was very uniform. Jiao et al. developed a nanosecond laser texturing technique to fabricate periodic surface structures, including dimples and grooves on Zr-based BMG surfaces [37]. They also investigated the effect of laser surface texturing on the wettability [15] and cytocompatibility [38] of the BMG surfaces. The modification of the surface wettability could be attributed to the laser-induced surface roughness and alteration of surface chemistry, and the enhanced cytocompatibility of the groove-textured BMG resulted from the combined effects of surface chemistry, wettability, and roughness. Du et al. fabricated laser-induced periodic surface structure (LIPSS) and nanoparticle structures on four types of Zr-based BMGs using femtosecond laser irradiation [40]. The experimental results indicated that the femtosecond laser nanostructured Zr-based BMG surface could lead to a distinct decrease in bacterial adhesion compared with the polished surfaces, which was strongly related to the laser-induced surface morphology and wettability. Although the above-mentioned research efforts have effectively modified the surface properties of BMG and achieved improved surface functionalities, none of them has attempted to realize the precise control of the key surface functions of BMG, e.g., control of surface wettability and microhardness, which could be more attractive and challenging, and also help to meet more different applications [43]. Further exploration of a time-efficient and cost-effective laser-based technique to realize the control of surface functionalities on the BMG surface is still of particular interest for the surface engineering community. 

Previously, the authors’ group has developed a facile nanosecond laser-based surface texturing method to achieve switchable wettability control of titanium alloy [36]. In this work, this laser-based surface texturing technique was further extended to modulate surface wettability and hardness on the BMG substrate. The periodic surface textures were directly created via laser texturing, and the surface chemistry was effectively controlled via heat treatment. The surface wettability was shown to convert from superhydrophilicity immediately upon lase texturing to superhydrophobicity after heat treatment, and the surface microhardness was significantly enhanced on the laser-induced surface texture. The underlying processing mechanisms were elucidated using scanning electron microscopy (SEM), energy-dispersive X-ray spectroscopy (EDAX), and X-ray photoelectron spectroscopy (XPS). Compared with the existing research works on laser texturing for functional surfaces, the laser-based surface texturing technique developed in this work proposed a novel and highly efficient approach to modulate and control the surface functionalities, which was achieved by the combination of high-speed UV nanosecond laser surface texturing and subsequent heat treatment. It is expected that the developed technique could provide a viable solution for the surface modification of Zr-based metallic glass, thus rendering a series of applications in the industrial and biomedical fields.

## 2. Materials and Methods

### 2.1. Materials

Commercially available Zr-based bulk metallic glass Vitreloy 1 with the nominal elemental composition Zr_41.2_Ti_13.8_Cu_12.5_Ni_10_Be_22.5_ (at%, purchased from METALLAB, Changzhou, China) was used in this work due to its excellent mechanical properties. The samples were mechanically grinded using grid SiC papers and further machined into thin sheets with a dimension of 30 mm × 30 mm × 2 mm using wire electrical discharge machining. Then, before laser surface texturing experiments, they were ultrasonically cleaned with acetone, ethanol, and deionized water successively to remove the contaminants. 

### 2.2. Laser-Based Surface Texturing

**Figure 1:**
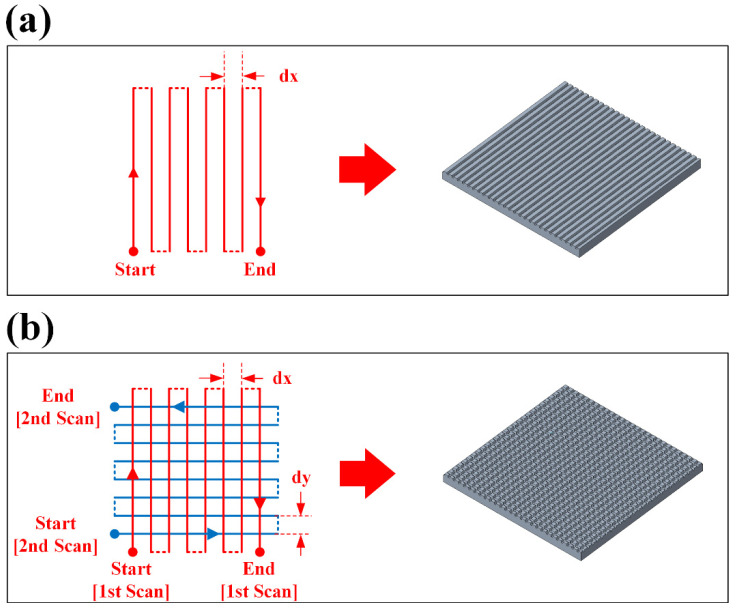
Pattern designs used for laser surface texturing of BMG: (**a**) unidirectional; (**b**) cross-hatch.

**Figure 2:**
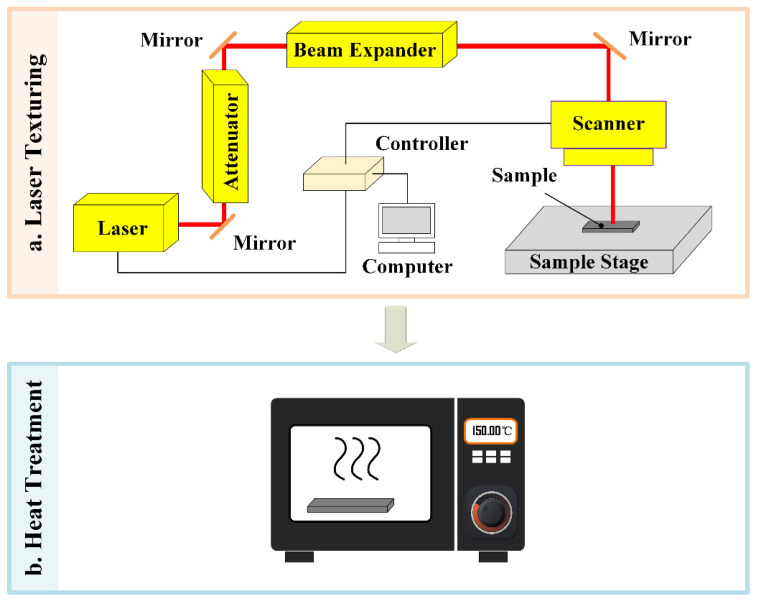
Schematics for the laser-based surface texturing experiments of BMG: (**a**) laser surface texturing; (**b**) heat treatment.

The laser-based surface texturing experiment mainly consists of two steps: (1) laser surface texturing and (2) heat treatment. Laser surface texturing experiments employed a laser marking machine (TH-UV200A, Tianhong Laser, Suzhou, China) equipped with a 355 nm UV laser source (AWAVE 355-15W-30K, Advanced Optowave, Ronkonkoma, NY, USA). The laser source emits a laser beam guided by reflective mirrors. The intensity of laser power is controlled by the attenuator, and the diameter of the laser beam can be expanded by the beam expander. The focusing lens of the system provides a focal spot diameter of ~35 µm during the laser texturing experiments. Unidirectional line pattern and cross-hatch pattern were used due to the ease and high efficiency of fabrication, as shown in Figure 1a,b. A series of preliminary experimental trials was attempted, and the optimal laser processing parameters were determined and utilized in this work, which can be found in Table 1. The schematics for the laser surface texturing experiments are shown in Figure 2a.

Immediately upon laser surface texturing, the laser textured BMG surfaces became superhydrophilic. To achieve the wettability transition, the laser textured BMG surfaces were placed in a conventional furnace for low-temperature annealing treatment, as shown in Figure 2b. An annealing temperature of 150 °C and a treatment duration of a maximum of 2 h were used during the heat treatment. 

### 2.3. Surface Characterizations

The surface morphology of the laser textured BMG surfaces was analyzed using field emission scanning electron microscopy (FESEM, Navo Nano SEM450, Hillsboro, OR, USA). The surface chemistry of the laser textured surface was evaluated using energy dispersive X-ray analysis (EDAX, FEI Sirion, Hillsboro, OR, USA) and X-ray photoelectron spectroscopy (XPS, PREVAC, Rogów, Poland). To examine the surface wettability of the laser textured surfaces, a contact angle goniometer (SDC-200, Sindin Precision Instrument Co., Ltd., Dongguan, Guangdong, China) equipped with a high-resolution CMOS camera was utilized to measure the static water contact angle value (θ_w_) on each surface. A water droplet with the volume of ~4 µL was dripped onto the sample surface using a digital syringe during each measurement. The optical image of the water contact angle measurement was captured using the CMOS camera, and image analysis software was utilized to determine the θ_w_ value for each measurement. For each sample, four θ_w_ measurements were performed at various locations, and the averaged θ_w_ value was reported. The standard deviation for each averaged θ_w_ measurement result was also calculated and added. The microhardness test was carried out using a Vickers hardness machine (HXD-1000TMSC/LCD, Shanghai, China) with a test load of 300 gf and a dwell time of 10 s. Similarly, five microhardness measurements were performed at different locations on the laser textured surface, and the averaged hardness value as well as the standard deviation for each measurement result were reported.

## 3. Results

### 3.1. Surface Morphology

**Figure 3:**
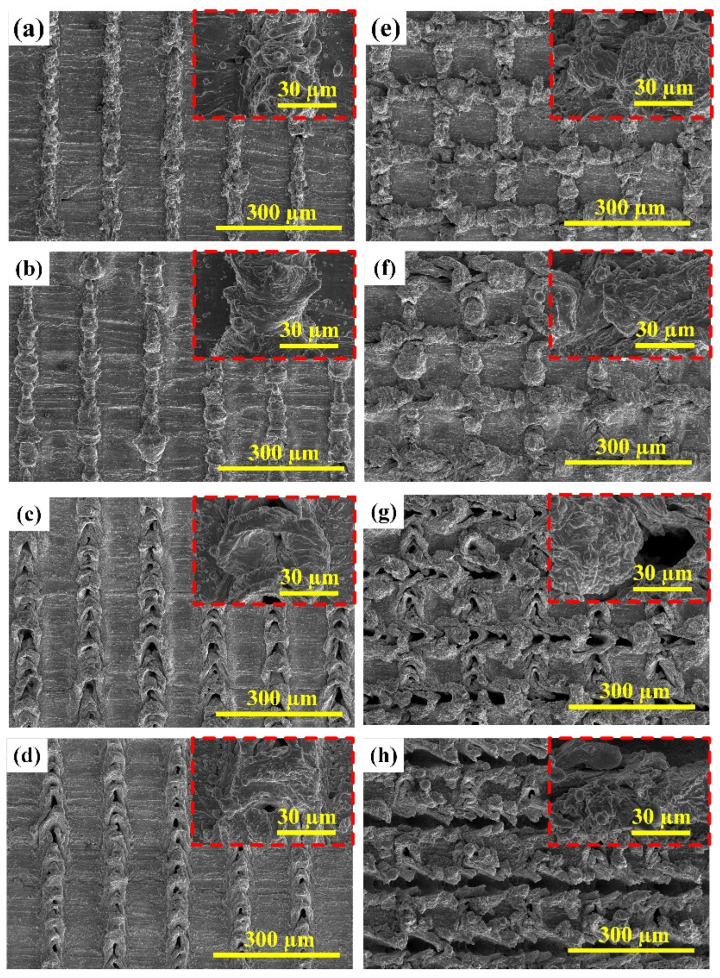
SEM micrographs of the laser textured BMG surfaces using different scanning speeds: (**a**–**d**) unidirectional surface patterns with the scanning speeds of 20 mm/s, 40 mm/s, 60 mm/s, and 80 mm/s; (**e**–**h**) cross-hatch surface patterns with the scanning speeds of 20 mm/s, 40 mm/s, 60 mm/s, and 80 mm/s.

The surface morphology of the laser textured BMG surface using different scanning speeds was examined by SEM, as shown in Figure 3. The SEM micrographs of the laser textured BMG surfaces with unidirectional surface patterns and various scanning speeds can be found in Figure 3a–d, while the SEM micrographs of the laser textured BMG surfaces with cross-hatch surface patterns and various scanning speeds are shown in Figure 3e–h. It is clearly seen that as the laser beam irradiated and ablated the BMG substrates, periodic micro-scale bulge structures were formulated as a result of the strong interaction between the laser beam and the BMG substrates. The SEM micrographs with high magnification indicate that there also have been some sub-micron or nano-scale particles covered on the micro-bulge structures, which can be mainly attributed to the ejection and deposition of nanoparticles during the laser-material interaction. In addition, it can be found that the laser-induced periodic surface structure exhibited distinct differences as the scanning speed increased. When the scanning speed of 20 mm/s was used, solid and defect-free micro-bulge structures were formed on the laser textured BMG surfaces with both unidirectional and cross-hatch patterns. As the scanning speed further increased, the surface structure gradually changed, which was illustrated by the variation of the micro-bulge height and the appearance of concave sections. Especially when the scanning speeds reached 60 mm/s and 80 mm/s, the concave structure became more distinct along the laser scanned line profiles for both surface patterns. The difference of the surface structure formation when varying the scanning speed should result from the time duration of laser–material interaction. When the lower scanning speed was utilized, the laser beam travelled relatively slowly along the scanned profile, and the number of pulses per irradiation point is higher, which would ensure adequate interaction between the laser beam and the BMG substrate and facilitate the formation of solid micro-bulge structures [44]. However, as the scanning speed increased, the laser beam moved faster and resulted in the decrease in the number of pulses per irradiation point. This would significantly weaken the impact of the laser beam on the BMG substrate. Consequently, due to the insufficient interaction time between the laser beam and the BMG substrate, fewer sub-micron or nano-scale particles and concave structures occurred on the laser textured surface with higher scanning speeds. 

**Figure 4:**
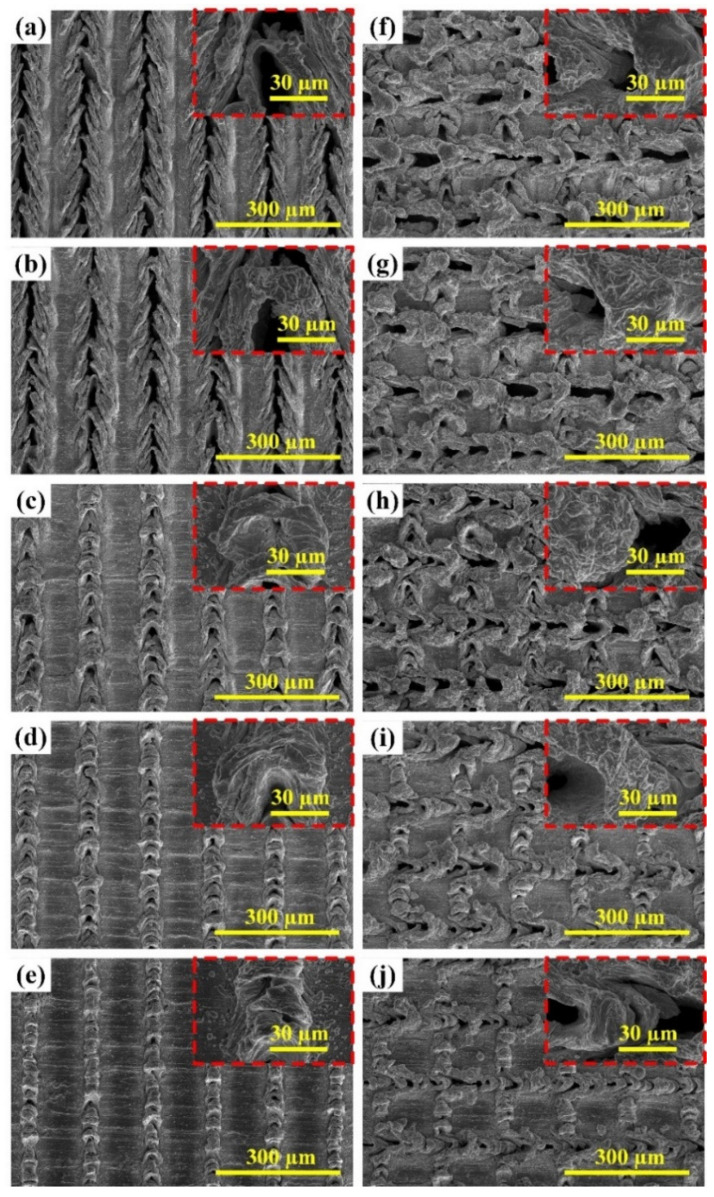
SEM micrographs of the laser textured BMG surfaces using different powers: (**a**–**e**) unidirectional surface patterns with the laser powers of 15.0 W, 13.5 W, 12.0 W, 10.5 W, and 9.0 W; (**f**–**j**) cross-hatch surface patterns with the laser powers of 15.0 W, 13.5 W, 12.0 W, 10.5 W, and 9.0 W.

The effect of laser power on the surface morphology of the laser textured BMG surface was further examined by SEM, as shown in Figure 4. The SEM micrographs of the laser textured BMG surfaces with unidirectional surface patterns and various laser powers can be found in Figure 4a–e, while the SEM micrographs of the laser textured BMG surfaces with cross-hatch surface patterns and various laser powers are shown in Figure 4e–h. From the SEM micrographs, it is evident that laser power can dramatically affect the formation of laser-induced structures on the BMG surface. By using higher laser powers (15.0 W and 13.5 W), clear concave microgrooves formed on the BMG surface, demonstrating strong vaporization of the material during the laser surface texturing process. As the laser power decreased (12.0 W and 10.5 W), the microgroove structure became less pronounced, only with some holes left on the laser scanned profiles. This indicated that less material evaporated from the substrate, and there appeared to be a restructuring on the laser textured surface. As the laser power kept decreasing (9.0 W), the concave structure almost disappeared, and the convex micro-bulge structure dominated. Similar trends could be observed for the laser textured BMG surfaces with both unidirectional and cross-hatch surface patterns. As clearly pointed out by Feng et al. [45,46], the laser-induced surface structure is a key factor that will affect the surface functionality, i.e., wettability, reflectivity, and hardness, and the laser processing parameters must be carefully chosen to ensure the formation of proper laser-induced structure, thus enabling the realization of desirable surface functionalities.

### 3.2. Surface Wettability

**Figure 5:**
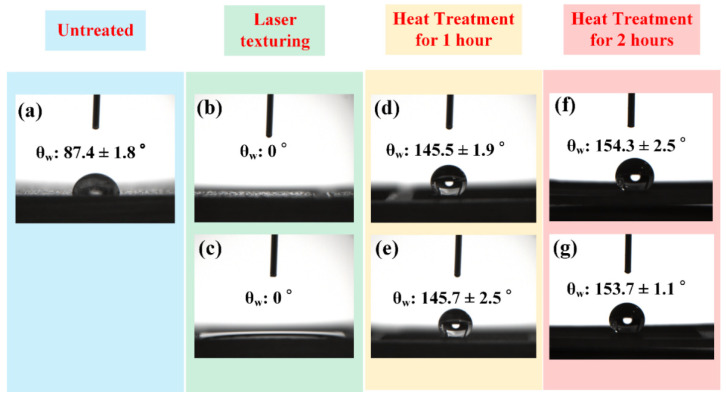
Contact angle measurement results for (**a**) untreated BMG surface; (**b**) laser textured BMG surface using the power of 12 W, the repetition rate of 30 kHz, the scanning speed of 40 mm/s, and a unidirectional surface pattern; (**c**) laser textured BMG surface using the power of 12 W, the repetition rate of 30 kHz, the scanning speed of 40 mm/s, and a cross-hatch surface pattern; (**d**,**e**) laser textured BMG surfaces in (**b**,**c**) after heat treatment for 1 h; (**f**,**g**) laser textured BMG surfaces in (**b**,**c**) after heat treatment for 2 h.

Surface wettability of the BMG surfaces with different surface patterns and treatment methods were evaluated via contact angle measurements. It can be found that the untreated BMG surface exhibited a θ_w_ of 87.4 ± 1.8°, as shown in Figure 5a, indicating that the untreated BMG surface is hydrophilic. Immediately upon laser surface texturing using the processing parameters of a laser power of 12 W, a repetition rate of 30 kHz, and a scanning speed of 40 mm/s, the laser textured BMG surfaces with both unidirectional (Figure 5b) and cross-hatch (Figure 5c) surface patterns exhibited a θ_w_ of 0°. This clearly indicates that the laser textured BMG surfaces are in a saturated Wenzel regime when being treated in the oxygen-containing atmosphere, which agrees well with the experimental results in [47]. After heat treatment for 1 h, the θ_w_ measurements indicated that the laser textured BMG surfaces with unidirectional and cross-hatch patterns exhibited θ_w_ values of 145.5 ± 1.9° and 145.7 ± 2.5°, respectively, indicating that a 1 h heat treatment rendered the laser textured BMG with high hydrophobicity. With a 2 h heat treatment, the laser textured BMG surfaces with both unidirectional and cross-hatch patterns became superhydrophobic, with θ_w_ values of 154.3 ± 1.9° and 153.7 ± 1.1°, respectively.

**Figure 6:**
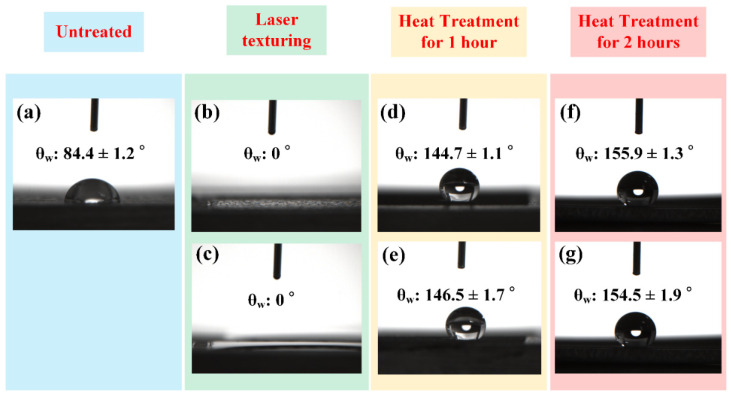
Contact angle measurement results for (**a**) untreated BMG surface; (**b**) laser textured BMG surface using the power of 10.5 W, the repetition rate of 30 kHz, the scanning speed of 60 mm/s, and a unidirectional surface pattern; (**c**) laser textured BMG surface using the power of 12 W, the repetition rate of 30 kHz, the scanning speed of 40 mm/s, and a cross-hatch surface pattern; (**d**,**e**) laser textured BMG surfaces in (**b**,**c**) after heat treatment for 1 h; (**f**,**g**) laser textured BMG surfaces in (**b**,**c**) after heat treatment for 2 h.

Figure 6 shows θ_w_ measurement results for the laser textured BMG with varied laser powers and scanning speeds. As shown in Figure 6a, the untreated BMG surface showed a hydrophilic nature with a θ_w_ of 84.4 ± 1.2°. For the laser texturing experiments, the following laser processing parameters were utilized: a laser power of 10.5 W, a repetition rate of 30 kHz, and a scanning speed of 60 mm/s. The θ_w_ measurement results (Figure 6b,c) confirmed the saturated Wenzel regime for the laser textured surfaces with both of the unidirectional and cross-hatch patterns. Subsequently, it can be found that a 1 h heat treatment turned the laser textured surfaces with both patterns highly hydrophobic, with θ_w_ values of 144.7 ± 1.1° and 146.5 ± 1.7°, respectively (Figure 6d,e). A 2 h heat treatment ensured that both of the laser textured surfaces reached superhydrophobicity, with θ_w_ values of 155.9 ± 1.2° and 154.5 ± 1.9°, respectively (Figure 6f,g). As discussed in the previous section, low-temperature heat treatment with an annealing temperature of ~150 °C has been proved to be an efficient approach to achieve wettability transition from superhydrophilicity to superhydrophobicity for various metallic materials such as aluminum, copper, stainless steel, and titanium [28,29,30,31,32,33,34,35,36]. However, this wetting state transition approach has never been utilized and confirmed for BMG. To the authors’ best knowledge, this work represents the first attempt to achieve wettability transition from superhydrophilicity to superhydrophobicity on BMG surfaces using the facile laser surface texturing technique combined with low-temperature annealing. The underlying processing mechanisms associated with the wettability will be explained in detail in the following sections.

### 3.3. Surface Chemistry

**Figure 7:**
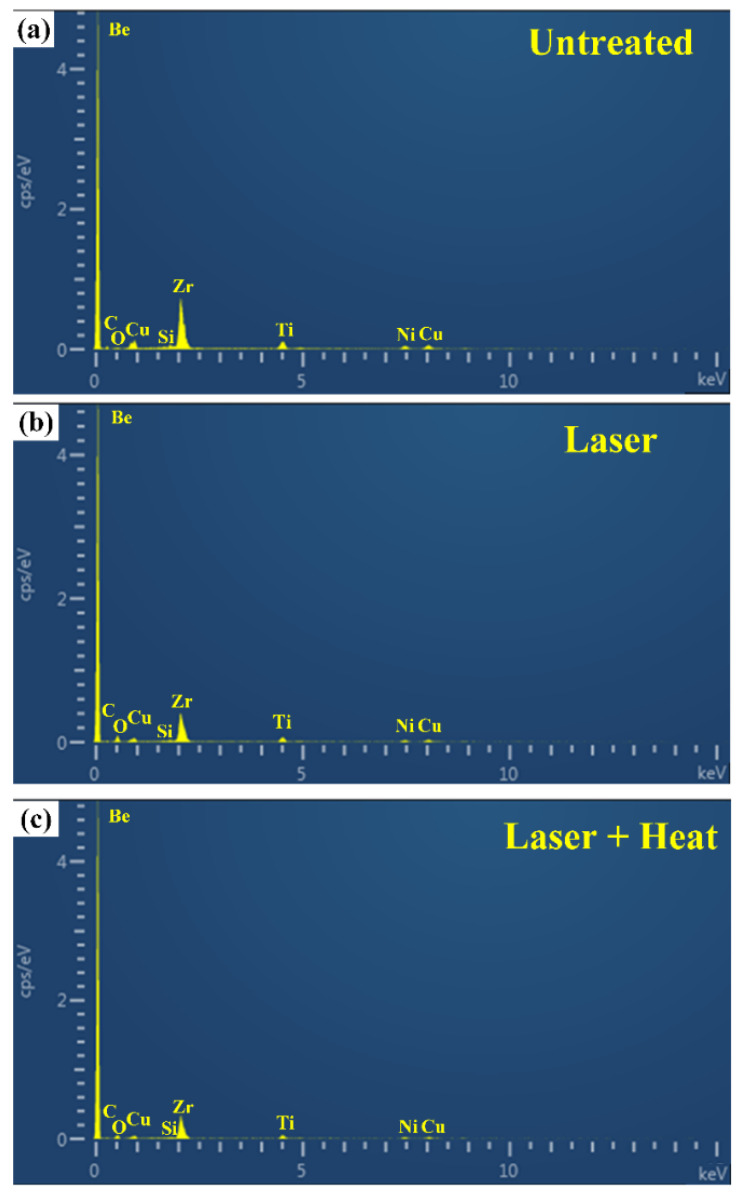
EDAX spectra for (**a**) untreated BMG surface; (**b**) laser textured BMG surface; (**c**) laser textured BMG surface followed by heat treatment.

Farasi et al. [48] pointed out that the surface chemical composition of metallic materials can be modified via material vaporization and oxidation during laser surface texturing, and Ngo et al. [31,49] believed that the increase in carbon content during heat treatment indicates the deposition of more hydrophobic functional groups on the laser textured surface, thus leading to the transition of surface wettability. Therefore, to explore and understand the variations of chemical compositions on the BMG surface and to further correlate surface wettability with surface chemistry, the laser textured BMG surfaces before and after heat treatment were analyzed by EDAX, as shown in Figure 7. The laser textured surfaces (with and without heat treatment) considered for the surface chemistry analysis were treated with the average laser power of 12.0 W, repetition rate of 30 kHz, pulse energy of 0.4 mJ, and power intensity of 2.08 GW/cm^2^. From Figure 7, it can be clearly seen that all the core elements can be detected on the three different types of surfaces. While compared with the untreated surface, the intensity of the Zr peak for the laser textured surface decreased, which is mainly a result of material vaporization during laser texturing. In the meantime, the O peak shows a notable increase in the laser textured surface compared with that of the untreated surface, indicating the BMG surface has been oxidized along with the formation of a periodic surface structure. As indicated in [50], as the electronic structure of metal oxide facilitates the formation of hydrogen bonds and increases the surface energy, the laser textured surface typically exhibited superhydrophobicity. For the laser textured surface with heat treatment, subtle increases in C and Si peaks can be detected, indicating that hydrophobic functional groups, including –CH_2_–, –CH_3_, C=C as well as thin PDMS layer, should have been absorbed and deposited onto the laser textured BMG surface, rendering the heat-treated surface with superhydrophobicity [33,49].

**Figure 8:**
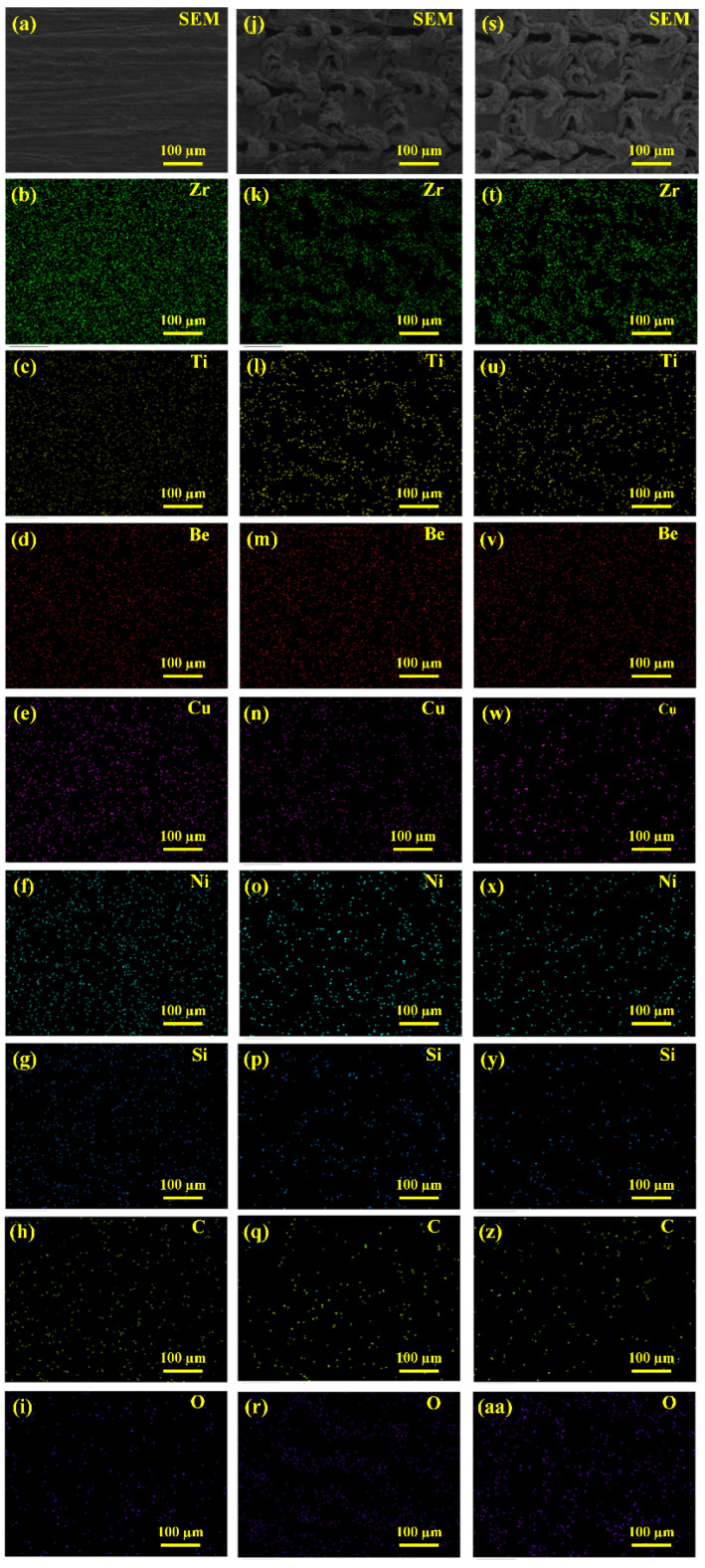
SEM/EDAX element mapping for (**a**–**i**) untreated BMG surface; (**j**–**r**) laser textured BMG surface; (**s**–**aa**) laser textured BMG surface followed by heat treatment. (**a**,**j**,**s**) are SEM micrographs representing the corresponding analyzed regions; (**b**–**i**,**k**–**r**,**t**–**aa**) are the EDAX maps showing the qualitative elemental distributions of Zr, Ti, Be, Cu, Ni, Si, C, and O.

The EDAX mapping data were also obtained to reveal the elemental distribution of all the core elements (Zr, Ti, Be, Cu, Ni, Si, C, and O) on each surface, as shown in Figure 8. From EDAX mapping, it can be clearly observed that all the core elements were uniformly distributed on the untreated surface. For the laser textured surfaces, more black areas can be observed, suggesting a reduction of the Zr, Ti, Be, Cu, and Ni elements, corresponding to the material removal during laser surface texturing. Distinct increases for the amount of O can be seen on the EDAX mapping as well, which strongly supports the oxidation during laser surface texturing. For the elemental distribution of C and Si, no clear change can be observed, which will be further examined using XPS.

**Figure 9:**
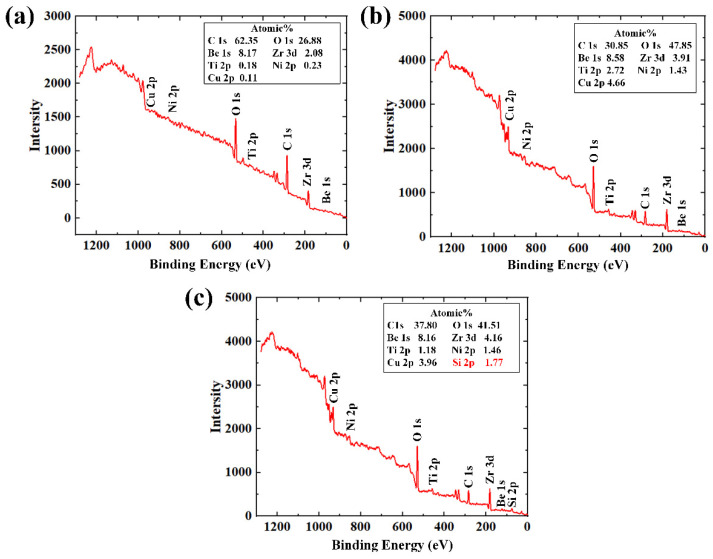
XPS full spectra of (**a**) untreated BMG surface; (**b**) laser textured BMG surface; (**c**) laser textured BMG surface followed by heat treatment.

Given the fact that EDAX is mainly a qualitative analysis method that could not provide conclusive results for the evaluation of the BMG substrate after laser texturing and heat treatment, XPS analysis was further employed to investigate the chemical changes on the surfaces that have been tested by EDAX. The XPS full spectra of the BMG surfaces considered for EDAX analysis and the corresponding atomic percentage of different elements are shown in Figure 9. It can be seen from the XPS full spectra of the untreated BMG surface (Figure 9a) that carbon and oxygen were detected as the major elements, which can be attributed to the contamination and oxidation of the BMG surface. For the constituent elements of the BMG Vitreloy 1 used in this work, small peaks of Zr 3d and Be 1s were observed on the untreated BMG surface, while the content of other elements (Ti 2p, Ni 2p, and Cu 2p) was negligible. Upon laser texturing, the atomic percentage of oxygen increased from 26.88% to 47.85% (Figure 9b). This clearly indicates that the laser textured BMG surface has been oxidized along with the formation of periodic surface structure during laser texturing. As the hydrogen bonds tend to form on the metal oxide, which also increases the surface energy [50], the laser oxidized BMG surface exhibited superhydrophilicity. After heat treatment, a clear atomic percentage increase in carbon from 30.85% to 37.80% can be observed on the laser textured BMG surface with heat treatment (Figure 9c). The increase in carbon content demonstrated that several different types of functional groups that exhibit hydrophobicity could have been absorbed onto the laser textured BMG surface during heat treatment, which rendered the heat-treated BMG surface highly hydrophobic [33,49]. In addition, the element Si was also detected on the heat-treated BMG surface, as shown in Figure 9c. The appearance of Si might represent the formation of a thin Si-based PDMS layer on the laser textured BMG surface during heat-treatment, which further increased the hydrophobicity of the laser textured BMG surface. The experimental findings in this work agree well with the results of Ti alloy using a similar method [36], and it is believed that the increase in carbon and the appearance of a thin PDMS layer contribute to the superhydrophobicity of the laser textured BMG surface after heat treatment.

**Figure 10:**
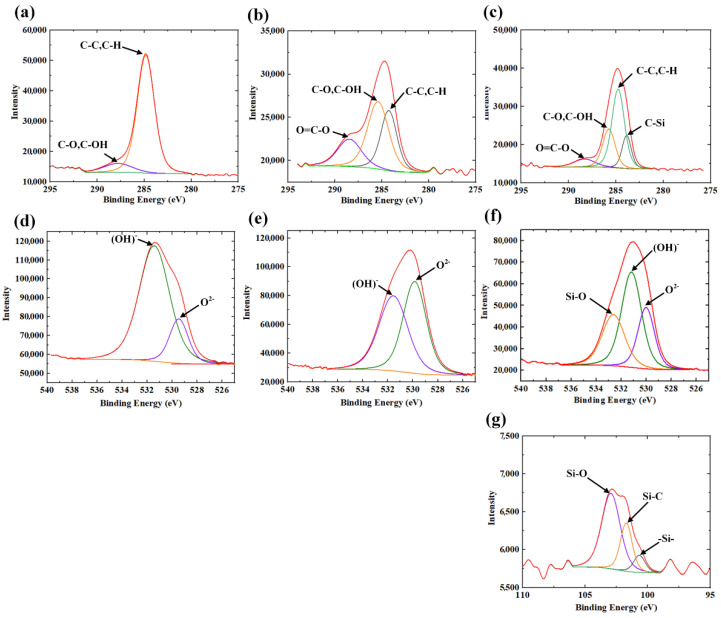
The high-resolution core elemental spectra of carbon on (**a**) untreated BMG surface; (**b**) laser textured BMG surface; (**c**) laser textured BMG surface followed by heat treatment. The high-resolution core elemental spectra of oxygen on (**d**) untreated BMG surface; (**e**) laser textured BMG surface; (**f**) laser textured BMG surface followed by heat treatment; and (**g**) the high-resolution core elemental spectra of silicon on the laser textured BMG surface followed by heat treatment.

Core elemental analyses were also performed for C, O, and Si elements, as shown in Figure 10. The core elemental analysis for C element can be found in Figure 10a–c. In Figure 10a, it is clearly shown that C element can be detected, confirming the contamination of the untreated BMG surface. For the laser textured BMG surfaces, it can be observed in Figure 10b and c that after heat treatment, the proportion of C–C and C–H peaks significantly increased, which helped to verify that hydrophobic functional groups with nonpolar C–C or C–H bonds should have been deposited onto the heat-treated BMG surface, inducing hydrophobicity on the surface. The core elemental analysis for O is shown in Figure 10d–f. It is clear that functional groups of (OH)^−^ and O^2−^ were detected on all of the BMG surface, while the proportion of O^2−^ on the laser textured BMG surfaces both with and without heat treatment was higher than that of the untreated BMG surface, proving that oxidation occurred during the laser texturing process. Finally, Figure 10g shows the core elemental analysis for Si element, revealing the appearance of the peaks for three functional groups Si–O, Si–C, and –Si– on the laser textured BMG surface after heat treatment. This should be attributed to the thin PDMS layer deposited on the laser textured BMG surface during heat treatment, which is derived from the silicone component on the furnace used in this work, as indicated in [32]. The underlying mechanism will be explained with details in the following section.

### 3.4. Surface Microhardness

**Figure 11:**
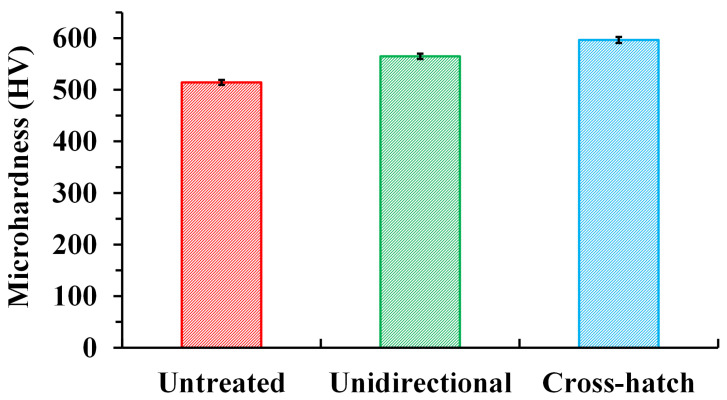
Microhardness measurements for untreated surface, laser textured BMG surface with unidirectional pattern, and laser textured BMG surface with cross-hatch pattern.

The microhardness measurement results of the untreated BMG surface, the laser textured BMG surface with unidirectional pattern, and the laser textured BMG surface with cross-hatch pattern can be found in Figure 11. The laser textured surfaces considered for the surface microhardness analysis were treated using the same processing parameters as those used for surface chemistry analysis. The experimental results indicated that the microhardness of the untreated BMG surface was 514.4 ± 4.9 HV. By laser surface texturing using the unidirectional surface pattern, the microhardness of the textured surface was increased to 564.9 ± 5.2 HV, representing an increase rate of 9.8% compared with the untreated surface. Meanwhile, the microhardness of the laser textured BMG surface with the cross-hatch pattern was further enhanced up to 596.7 ± 5.8 HV, indicating an increase rate of 16.0% compared with the untreated surface. As evident from the SEM micrographs shown in Figure 3 and Figure 4, the laser surface texturing process created a combination of micro-scale, sub-micron, and nano-scale structures on the BMG substrate, which have generated much finer grains on the surface. As indicated by the Hall–Petch relationship, finer grains will result in higher mechanical strength [51,52]. More grain boundaries can be generated using finer grains, and the resistance to hinder dislocation motion will increase, leading to the increase in surface microhardness. In addition, compared with the laser textured surface with the unidirectional pattern, one more laser scan from the vertical direction would significantly increase the density of surface structures at all scales on the BMG substrate, which would contribute to the further enhancement of microhardness [53]. The microhardness measurement results indicate that the developed laser-based surface texturing process can effectively enhance the surface mechanical strength of the BMG substrate.

### 3.5. Processing Mechanism Analysis

**Figure 12:**
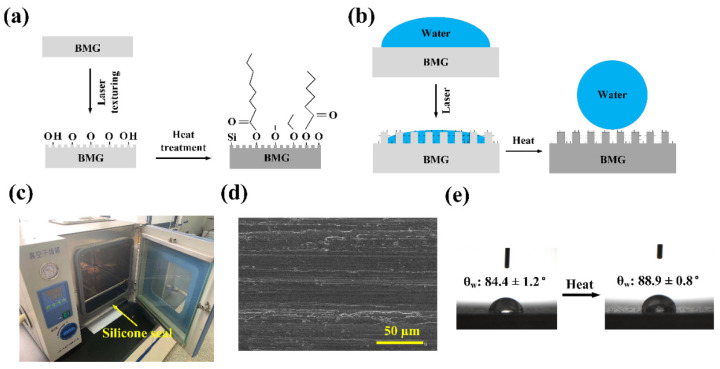
Schematic for the mechanism of wettability transition for the laser textured BMG surface after laser texturing and heat treatment: (**a**) alteration of surface chemistry; (**b**) alteration of surface wettability; (**c**) demonstration of the silicone seal on the furnace used in this work; (**d**) SEM micrograph of the untreated BMG surface; (**e**) contact angle measurement results for the untreated BMG surface before and after heat treatment.

The underlying mechanism for the wettability transition of BMG surface during laser texturing and heat treatment is schematically depicted in Figure 12. As shown in Figure 12a, nanosecond laser surface texturing not only generated periodic surface structures on the BMG substrate but also oxidized the surface in the ambient air with the existence of water vapor [35,47]. Consequently, formation of oxide and hydroxide layers would occur simultaneously on top of the BMG substrate, which will drastically increase the surface energy and lead to superhydrophilicity [28,54]. During heat treatment, the wettability transition from superhydrophilicity to superhydrophobicity can be ascribed to two aspects: on the one hand, it is proposed that the organic compounds existed in air with nonpolar C–C and C–H bonds that have been absorbed onto the laser-induced micro-bulge or groove structures, and this process has been accelerated as the heat treatment temperature increased. The heat treatment helped to eliminate the hydrophilic –OH functional group from the laser textured BMG surface and created more active sites on the surface for the subsequent absorption of organic compounds [55]. As a result, more hydrophobic functional groups, such as –CH_2_– and –CH_3_, have been attracted onto the laser textured BMG surface, leading to the increase in surface hydrophobicity. On the other hand, the XPS analysis confirmed the existence of Si element on the laser textured BMG surface, indicating that a thin PDMS layer could have been deposited on the surface. The source of the Si element was originated from the silicone seal on the furnace, which was partially vaporized and deposited onto the laser textured BMG surface, as shown in Figure 12c. Since the silicon-based organic polymer PDMS is known to be hydrophobic, it could help to further enhance the hydrophobicity of the laser textured BMG surface, leading to superhydrophobicity eventually. It is thus believed that absorption of hydrophobic airborne organic compounds and generation of the thin PDMS layer both contributed to the surface wettability transition on the laser textured BMG surface during heat treatment. Therefore, from an initial contact angle of ~85° on the untreated BMG surface, the surface wettability was converted to superhydrophilicity (~0°) after laser texturing, and superhydrophobicity (>150°) after heat treatment, as shown in Figure 12b.

In the meantime, although surface chemistry plays a key role for the wettability transition, it is proposed that the laser-induced surface structure is equally critical for achieving the target wettability condition. This could be verified by what has been observed on the untreated BMG surface before and after heat treatment. Figure 12d shows the SEM micrograph of the untreated BMG surface, indicating that there are only grinding marks without any distinct periodic surface structures. The contact angle measurement results indicated that the initial θ_w_ was 84.4 ± 1.2° on the untreated BMG before heat treatment, and the θ_w_ was measured to be 88.9 ± 0.8° after heat treatment. This clearly indicates that alteration of surface chemistry alone could not render the BMG material with superhydrophobicity. Surface structure and surface chemistry should be modulated and controlled spontaneously to achieve the desired surface functionality.

## 4. Conclusions

In this work, a facile and efficient laser-based surface texturing method was developed to modulate and control the surface functionalities of Zr-based BMG. The following findings can be summarized:

(1) The developed laser-based surface texturing technique consists of two steps: laser texturing and heat treatment. Laser texturing generated the periodic surface structures and oxidized the BMG surface, while the subsequent heat treatment accelerated the absorption of hydrophobic airborne organic compounds and deposited a thin PDMS layer on the laser textured BMG surface.
(2) It is found that the untreated BMG surface is hydrophilic with a θ_w_ of 84.4 ± 1.2°. Immediately upon laser texturing, the laser textured BMG surface exhibited a θ_w_ of 0°, indicating the surface turned superhydrophilic. After a heat treatment of 2 h, the laser textured BMG surface became superhydrophobic with a θ_w_ higher than 150°.
(3) Through careful experimental validation and analysis, it is believed that the laser-induced surface texture and modified surface chemistry are equally important for achieving the desirable wettability condition.
(4) The microhardness of the laser textured BMG surface is also notably increased due to the higher microstructure density and more grain boundaries generated on account of the laser surface texturing process.

This method will provide a feasible and highly efficient solution for regulating and controlling the surface functionalities of BMG, which will render more practical and important applications.

## Figures and Tables

**Table 1:** Processing parameters for the laser-based surface texturing experiments.

Name of Parameter	Value
Average power (W)	9.0, 10.5, 12.0, 13.5, 15.0
Repetition rate (kHz)	30
Pulse width (ns)	20
Scanning speed (mm/s)	20, 40, 60, 80
Step size (µm)	150
Power intensity (GW/cm^2^)	1.56~2.60
Pulse energy (mJ)	0.3~0.5
Heat treatment temperature (°C)	150
Heat treatment duration (h)	2
